# Optimal TELSAM-Target Protein Linker Character is Target Protein-Dependent

**DOI:** 10.1063/4.0001133

**Published:** 2025-10-27

**Authors:** Maria Jose P Romo, Alihikaua Keliiliki, Jacob C Aerett, Joseph F Gonzalez, Ethan Noakes, Elijah W Wilson, Conrad Smith, Blake Averett, Dalton Hansen, Riley Nickles, Miles Bradford, Sara Soleimani, Tobin Smith, Supeshala Nawarathnage, Prasadika Samarwickrama, Ariel Kelsch, Derick Bunn, Cameron Stewart, Seth Brown, Tzanko I Doukov

**Affiliations:** 1 Brigham Young University, Provo, Utah, USA; 2 Stanford Synchrotron Radiation Lightsource, Menlo Park, California, USA

## Abstract

The fusion of the sterile alpha motif domain of the human translocation ETS leukemia protein (TELSAM) to a protein of interest has been shown to significantly enhance crystallization propensity and improve diffraction quality. TELSAM is a pH-dependent, polymer-forming protein crystallization chaperone which, when covalently fused to a protein of interest, forms a stable and homogenous crystal lattice. However, despite its success, the challenge persists in the significant impact of the linkers between TELSAM and the protein of interest on crystal and diffraction quality, with the best linker selection relying on trial-and-error methods thus far. To address this, we designed multiple constructs with several types of linkers—rigid (helical fusion), semi-rigid (Pro and Ala), and flexible (poly-glycine)—of varying lengths to connect the designed ankyrin repeat protein (DARPin) to TELSAM. Likewise, previous studies revealed that the 10xHis tag at the TELSAM N-terminus can either facilitate or hinder the ordered crystallization of target proteins attached via flexible or semi-rigid linkers. To address these challenges, we generated semi- rigid and flexible constructs with and without the 10xHis tag. Our findings indicate that short semi-flexible and rigid linkers consistently yield large crystals within 24 hours with a DARPin target protein, but that flexible linkers perform best with a TNK1 UBA domain target protein. Removing the 10xHis tag enhanced crystallization rates, improved crystal morphology, and increased the crystallization propensity of semi-flexible and flexible linker constructs. While removing the His tag did not have a significant effect on crystal size, it improved the diffraction limits and crystal quality of the 1TEL-PA-DARPin construct. These results suggest that the ideal linker selection primarily depends on the properties of the target protein. Our data support the recommendation to use a short yet flexible or semi-flexible linker between TELSAM and the target protein to facilitate protein crystallization and high-resolution structure determination.

